# Hit or Run: Exploring Aggressive and Avoidant Reactions to Interpersonal Provocation Using a Novel Fight-or-Escape Paradigm (FOE)

**DOI:** 10.3389/fnbeh.2017.00190

**Published:** 2017-10-17

**Authors:** Frederike Beyer, Macià Buades-Rotger, Marie Claes, Ulrike M. Krämer

**Affiliations:** ^1^Institute of Cognitive Neuroscience, University College London, London, United Kingdom; ^2^Department of Neurology, University of Lübeck, Lübeck, Germany; ^3^Institute of Psychology II, University of Lübeck, Lübeck, Germany

**Keywords:** approach, avoidance, aggression, provocation, retaliation

## Abstract

Interpersonal provocation presents an approach-avoidance conflict to the provoked person: responding aggressively might yield the joy of retribution, whereas withdrawal can provide safety. Experimental aggression studies typically measure only retaliation intensity, neglecting whether individuals want to confront the provocateur at all. To overcome this shortcoming of previous measures, we developed and validated the Fight-or-Escape paradigm (FOE). The FOE is a competitive reaction time (RT) task in which the winner can choose the volume of a sound blast to be directed at his/her opponent. Participants face two ostensible opponents who consistently select either high or low punishments. At the beginning of each trial, subjects are given the chance to avoid the encounter for a limited number of times. In a first experiment (*n* = 27, all women), we found that fear potentiation (FP) of the startle response was related to lower scores in a composite measure of aggression and avoidance against the provoking opponent. In a second experiment (*n* = 34, 13 men), we altered the paradigm such that participants faced the opponents in alternating rather than in random order. Participants completed the FOE as well as the Dot-Probe Task (DPT) and the Approach-Avoidance Task (AAT). Subjects with higher approach bias scores in the AAT avoided the provoking opponent less frequently. Hence, individuals with high threat reactivity and low approach motivation displayed more avoidant responses to provocation, whereas participants high in approach motivation were more likely to engage in aggressive interactions when provoked. The FOE is thus a promising laboratory measure of avoidance and aggression.

## Introduction

Aggressive behavior is a great challenge to individuals and to society. It is thus not surprising that research on aggression has been conducted for decades and continues to be an important problem addressed by scientific experiments today (Anderson and Bushman, 1997). Laboratory experiments on human aggression usually employ one of several well-established paradigms, which are derived from different theories of human aggression. One central aspect is interpersonal provocation: two of the most widely used aggression paradigms, the point-subtraction-aggression-paradigm (PSAP; Cherek, 1981) and the Taylor Aggression Paradigm (TAP; Taylor, 1967) confront the participant with an ostensible opponent, who in some way inflicts harm upon the participant. The participant then has the option to retaliate, which is directly measured within the paradigms. This approach is based on the well-established theory that reactive aggression is usually elicited as a response to some form of provocation or frustration (Anderson and Bushman, 1997; Lawrence, 2006). However, one shortcoming of these paradigms is that they largely limit the participant’s behavioral options to showing lower or higher levels of aggression. In real-life hostile situations, one usually has the option to avoid the aggressive interaction altogether and withdraw from the situation. Limiting the range of behavioral options possibly limits the applicability of laboratory findings to real-life aggression. Thus, implementing an escape option in laboratory aggression paradigms is an important step towards improving research on aggression (Tedeschi and Quigley, 1996).

In the PSAP, participants are led to believe that they will interact with another player in another room while trying to earn points, which can later be exchanged for money. The participant can earn points by pressing a button as quickly as possible. With another button, the participant can subtract points from his co-player. Provocation is implemented as the co-player’s subtraction of the participant’s points, whereas aggressive behavior is measured as the number of times the participant uses the point subtraction button to inflict cost upon his co-player. In some versions of the PSAP, a protective button is implemented, which protects the participant from point subtraction for a certain time (Cherek et al., 1997).

In the TAP, participants are also led to believe that they are competing against another player. Subjects are told they will engage in a reaction time (RT) competition with the opponent. They are required to respond quickly to a stimulus, and are led to believe that the faster player in each run wins. The loser gets punished with an aversive stimulus (e.g., a mild electric shock or a sound blast), which can be adjusted in intensity. In each trial, the winner determines the intensity of punishment for the loser. Provocation in this paradigm is manipulated as the punishment level assigned to the participant, whereas aggression is measured as the punishment level selected by the participant for the opponent.

These paradigms have been widely used in behavioral research on aggression and, more recently, also in research on the neural basis of aggressive behavior (Krämer et al., 2007; Lotze et al., 2007; Kose et al., 2015). In both paradigms, aggressive behavior is non-instrumental insofar as the main outcome of the task (i.e., winning the RT task; earning points) is not improved by aggressive behavior but may actually (in case of the PSAP) be hindered by it. One conceptual advantage of the TAP is that while in the PSAP aggression is costly (the participant cannot simultaneously subtract and earn points), in the TAP aggressive behavior can be measured independently of cost-benefit considerations. Similarly, while avoidance behavior is assumed to be related to fear (Carver, 2004), in the PSAP the protective button may be used based on considerations of monetary tradeoff (weighing the points missed while pressing the protective button against the points saved by avoiding subtraction). As such, it may constitute an imperfect model of real-life avoidance behavior.

This latter point, however, also poses an important limitation for the extrinsic validity of the TAP. We have previously argued (Beyer et al., 2014) that based on theories about the role of emotional reactivity in aggressive behavior, one would expect a negative relationship between fear reactivity and aggressive behavior in the TAP. Specifically, anger, as an approach-related affect (Carver and Harmon-Jones, 2009) should be most reliably elicited by provocation in participants low in fear reactivity and high in approach motivation. On the other hand, participants high in fear reactivity should react to a provocative confrontation with increased avoidance tendencies, rather than aggressive approach tendencies. In a functional magnetic resonance imaging (fMRI) study using the TAP, in which we measured threat reactivity as fear potentiation (FP) of the startle response, we found support for this hypothesis on a neural level (Beyer et al., 2014). The startle response in humans can be measured as the eye-blink amplitude in response to a sudden burst of white noise. FP is defined as the amplification of this amplitude when the participant is watching threatening rather than neutral pictures, and has been shown to be a good measure of emotional reactivity to threat (Vaidyanathan et al., 2009). In participants low in FP, we observed increased activity in areas of the so-called mentalizing network when they were confronted with a provocative opponent in the TAP. For participants high in FP, we observed the opposite effect, a reduction of activity in the mentalizing network due to provocation. The mentalizing network consists of cortical structures recruited when people take the perspective of another person in order to infer his/her thoughts, wishes or intentions (Lieberman, 2007). We therefore interpreted this effect as cognitive avoidance of the aggressive interaction in highly fearful participants (Beyer et al., 2014). However, we observed no effects on a behavioral level. One potential reason for this is the lack of an avoidance option in the TAP. Participants high in fear reactivity had no option of escaping the confrontation with the aggressive opponent and consequently may have adopted a tit-for-tat-like strategy. In many everyday incidents of provocation, however, avoidance is a realistic and valid behavioral option. Thus, we expect that the ecological validity of aggression paradigms should be increased by including a true avoidance option, producing the proposed relationship between personality traits (namely fear reactivity) and aggressive behavior.

In this study, we present a novel interactive aggression paradigm with an avoidance option: the “Fight-Or-Escape” (FOE) paradigm. In a first experiment, we implemented the task in a female student sample, also measuring FP of the startle response in a similar setup as we previously used for the TAP. This experiment is designed to test our previous interpretation of the non-existing relationship between FP and aggression in the TAP. To further validate our new paradigm, in a second experiment, we combined the task with two other well-established tests of social avoidance tendencies, the approach-avoidance-task (AAT; Roelofs et al., 2005) and a dot-probe-task (DPT; MacLeod et al., 1986) using emotional facial stimuli. We expected that fear reactivity and social avoidance should be associated with avoidant behavior and less aggression towards a provocative opponent.

## Experiment 1

### Materials and Methods

#### Participants and Procedure

Forty-three healthy female volunteers (M_age_ = 22 ± 2 years) participated in this study. We recruited only women in order to keep comparability with the previous study (Beyer et al., 2014). Participants were invited to the lab in groups of three. They were informed that two different experiments would be carried out: one EMG-measurement (the startle measure) and one group-task. The order of the two tasks was randomized across participants.

For the aggression task, the three participants received written instructions together. Prior to this task, participants filled out questionnaires assessing approach/avoidance tendencies and empathy (see below). After the aggression task, participants filled out a questionnaire probing for suspicion concerning the task, as well as a questionnaire on trait aggression. Finally, all participants were fully debriefed and reimbursed for their participation with 8 Euro per hour. Importantly, participants always received the same endowment regardless of their performance, and this was made clear to them before the measurement. This study was carried out in accordance with the recommendations of the University of Lübeck Ethics Commission (Ethikkommission der Universität zu Lübeck) with written informed consent from all subjects. All subjects gave written informed consent in accordance with the Declaration of Helsinki. The protocol was approved by the University of Lübeck Ethics Commission (Ethikkommission der Universität zu Lübeck).

#### Fight-or-Escape (FOE) Paradigm

The FOE-paradigm was set up as a competitive RT game for three people. Participants were instructed that each would randomly be assigned one of three characters from the “Lord of the Rings” trilogy (Tolkien, 1954). As a background story, participants were told that Sauron and Saruman were sending out orcs to obtain the Ring of Power from Frodo, and that during the game it would be decided whether Frodo succeeds in destroying the ring or whether it is taken by his opponents. The three available characters were Frodo, Sauron and Saruman. In fact, each participant was assigned the role of Frodo. Participants were instructed that Sauron and Saruman would compete together against Frodo.

**Figure 1:**
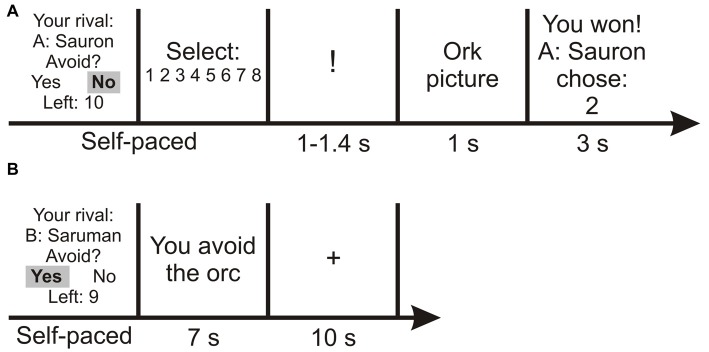
Example trials of the Fight-or-Escape (FOE) paradigm. **(A)** Trial in which the participant confronted the opponent. **(B)** Trial in which the participant avoided the opponent. The fixation cross is only depicted for the avoidance trial, but appeared regardless of the participant’s decision. See main text for details.

In each trial, Frodo would be playing against one of his two opponents and the winner of each trial would receive one point, with the points of Sauron and Saruman being summed together. At the end of the game, the party with the highest score (Frodo or Sauron and Saruman) would win. Participants were informed that the outcome would not affect their endowment. The winner of each trial was decided in a simple RT task: an exclamation mark was presented, followed by the picture of an orc. Frodo and his opponent had to respond to the orc by button press as quickly as possible. The faster player received one point, whereas the loser was punished with an aversive sound. At the beginning of each round, participants selected a punishment level (i.e., the noise level, ranging from 1 to 8) for their opponent, in case that the participant would win. Additionally, at the beginning of each trial, Frodo had the option of putting on the ring and thus becoming invisible, to avoid the confrontation. In that case, nobody received a point and nobody got punished; this choice constituted the “escape” option, i.e., avoiding potential punishment while foregoing the chance of earning a point. Thus, the sequence for one trial was as follows (Figure 1): (1) information on which opponent the participant was playing against; (2) choice of putting on the ring; in case Frodo put on the ring, a short message (“You escape the orc”) was displayed and the trial ended; (3) if Frodo did not put on the ring: punishment selection; (4) RT task; and (5) outcome phase: information on who won and which punishment level the opponent selected.

During screen number 2 (option of putting on the ring), Frodo was presented with the remaining number of times he could use the ring. Throughout the game, he could put on the ring a maximum number of 10 times. If a participant selected the ring after the 10 permitted escape options had been used, the message “You cannot use the ring anymore” was displayed and the trial then automatically proceeded to the punishment selection.

The game was programed such that subjects would win about 50% of trials against each opponent. Winning and losing was not related to RT, unless participants were slower than 500 ms, in which case they would lose. The behavior of Sauron and Saruman was programed such that one opponent was non-provocative, selecting low punishments (range 1–4, mean = 2.3), whereas the other was highly provocative, selecting high punishments (range 4–8, mean = 6.0). Punishments were randomly distributed across trials for each opponent. Frodo played 20 trials against each opponent in randomized order. Thus, Frodo had the option of avoiding up to 50% of the confrontations with the provocative opponent. Frodo could distribute using the ring between the two opponents in any ratio, and he was not obliged to use all 10 escape options. Since nobody received a point in escape trials, putting on the ring did not affect the ultimate outcome of the game. The identity of the provocative opponent (Sauron or Saruman) was randomized across participants. The intensity of the punishment (a Styrofoam scratching sound) was adapted to each participant’s tolerance before starting the task. After the task, participants rated the perceived unpleasantness of the loudest and lowest tone in a scale from 1 to 8.

#### Behavioral Measures

A range of behavioral measures can be derived from this task: pure aggression measures were obtained by calculating mean punishment selection for each opponent. However, this score would be identical for a participant who avoided the aggressive opponent in half the trials but otherwise retaliated with high punishment selections, and a participant who never avoided and behaved aggressively. Avoidance measures were obtained by counting the number of times the ring was used to avoid each opponent. Similar to the problem mentioned for the mean aggression score, this avoidance measure would be identical for participants high and low in punishment selections, if both avoid the same number of trials. To address these issues, we additionally calculated a combined aggression-avoidance score by summing punishment selections for each opponent across all trials. For this measure, avoidance trials are scored as zero. Consequently, this score is affected both by the number of times a participant chose not to play against an opponent, and by the punishment she selected when she did. Accordingly, this measure reflects the absolute amount of aggression shown towards the opponent. A medium score for the provocative opponent could be reached by a participant who frequently avoided him, but behaved aggressively in the remaining trials, or a participant who did not avoid him, but showed moderate levels of aggression.

#### Personality Questionnaires

A German version (Herzberg, 2003) of the Buss and Perry Aggression Questionnaire (AQ; Buss and Perry, 1992) was used to assess trait aggressiveness. The AQ consists of four subscales: physical aggression, verbal aggression, hostility and anger. To assess approach and avoidance tendencies, a German version (Strobel et al., 2001) of the behavioral inhibition and activation scales (BIS/BAS; Carver and White, 1994) was used. We also used the Interpersonal Reactivity Index (IRI; Davis, 1983) in our own translation to measure empathy and perspective taking tendencies.

#### Measurement of Fear Potentiation

To measure FP, we used a setup which was adapted from previous studies (Caseras et al., 2006; Conzelmann et al., 2009). Participants were presented with 51 pictures from the International Affective Pictures System (IAPS; Lang et al., 1999). Half of these pictures were threatening (e.g., a gun pointed at the viewer, an attacking dog), the other half were neutral (e.g., a secretary on the phone; household objects). Pictures were presented in a fixed order which was set up randomly with the constraint that no more than two pictures of the same valence were presented consecutively. Each picture was presented for 6 s with a 12 s inter trial interval (ITI), during which a white central cross was presented on a black background. During 18 threatening and 21 neutral pictures, a short burst of white noise (50 ms, 95 dB), was presented over speakers 1.5, 2.8 or 4.0 s after picture onset. For the remaining 12 pictures, the startle probe was presented during the ITI and these trials were not analyzed. To account for initial habituation of the startle response, four startle probes were presented while participants watched the fixation cross. Additionally, the first three picture trials (all neutral), were discarded.

#### EMG Measurement and Analysis

Two Ag-AgCl electrodes were placed below the left lower eyelid, one in line with the pupil and the other 1–2 cm to the left of the first. A ground electrode was positioned centrally on the forehead. Prior to electrode placement, the skin was treated with a peeling paste and alcohol. The EMG signal was amplified and recorded at a sampling rate of 250 Hz using an EEG amplifier (32-channel Brainamp; Brain Products).

We analyzed EMG recordings with EEGLAB, a MATLAB-based open-source toolbox (Delorme and Makeig, 2004). EMG signals were high-pass filtered at 10 Hz, low-pass filtered at 500 Hz and baseline-corrected using the 50 ms prior to onset of the startle probe as baseline. We then visually inspected each startle trial for artifacts. Trials with excessive noise or eyeblinks in the 50 ms baseline period were excluded. Blink magnitude was measured as the maximum absolute amplitude in an interval of 20–160 ms following the startle probe. Blink scores were z-transformed within each participant across all trials. We then subtracted the mean standardized blink amplitude for neutral pictures from the respective value for threatening trials, to get individual FP scores.

#### Statistical Analyses

We first conducted paired *t*-tests to compare mean aggression and mean avoidance scores for the provoking vs. non-provoking opponent, as well as mean blink amplitude for threatening vs. neutral pictures.

To investigate the relationship between FP and aggressive and avoidant behavior, we correlated FP scores with the respective behavioral measures (mean aggression, number of avoidance choices, sum of punishment selections across trials) for each opponent. We hypothesized that FP should be negatively related to aggressive behavior and positively to avoidant behavior, resulting in a negative correlation between FP and the summed punishment score. In the presence of a significant relationship, we *post hoc* compared correlation coefficients between opponents in R v1.3.1 with the *r.test()* function, available in the* psych* package v1.5.6 (Revelle, 2017). On an exploratory level, we also correlated personality questionnaire scores with FP and behavioral measures from the task. Significance was set in all cases at *p* < 0.05. Table 1 shows descriptive statistics for all measures in Experiment 1.

**Table 1:** Descriptive statistics for variables in Experiment 1 (*n* = 27).

	Mean ± SD
AQ total	56.12 ± 10.52
AQ physical aggression	16.80 ± 4.68
AQ physical aggression	10.60 ± 2.84
AQ verbal aggression	14.00 ± 3.23
AQ anger	14.52 ± 3.84
BIS	2.14 ± 0.50
BAS	1.82 ± 0.40
IRI fantasy scale	19.80 ± 3.75
IRI empathic concern	22.40 ± 2.16
IRI perspective taking	16.76 ± 1.83
IRI personal distress	16.44 ± 3.17
Startle neutral (Z)	−0.015 ± 0.13
Startle threatening (Z)	0.015 ± 0.12
FOE avoidance HP	3.30 ± 2.70
FOE avoidance LP	2.59 ± 2.25
FOE aggression HP	4.02 ± 1.64
FOE aggression LP	3.18 ± 1.53

*AQ, Aggression Questionnaire; BIS, Behavioral Inhibition System; BAS, Behavioral Activation System; IRI, Interpersonal Reactivity Index; Z, Z scores; HP, High Provocation; LP, Low Provocation*.

### Results

Of the 43 participants, 16 had to be excluded due to the following reasons: technical problems during startle measurements and/or bad EMG data quality (13); suspicion concerning the aggression task (3). Participants rated the highest tone (*M* = 5.8, SE = 1.8) as significantly more unpleasant than the lowest tone (*M* = 1.8, SE = 0.2), *t*_(26)_ = 11.1, *p* < 0.001.

On average, participants selected higher punishment levels for the provoking (*M* = 4.0, SE = 0.3) than the unprovocative opponent (*M* = 3.1, SE = 0.2), *t*_(26)_ = 3.2, *p* < 0.01. Of the 10 avoidance options, participants used on average 5.9 (SE = 0.7; range 0–10). There was no significant difference in the number of times participants avoided the provocative (*M* = 3.3; SE = 0.5) and non-provocative opponent (*M* = 2.5; SE = 0.4), *p* = 0.33. There was also no significant difference (*p* = 0.61) between startle responses to neutral (*M* = −0.015, SE = 0.13) and threatening pictures (*M* = 0.015, SE = 0.12). Across participants, however, there was great variability in FP (range −0.59 to 0.40; mean = 0.03; SE = 0.05).

**Figure 2:**
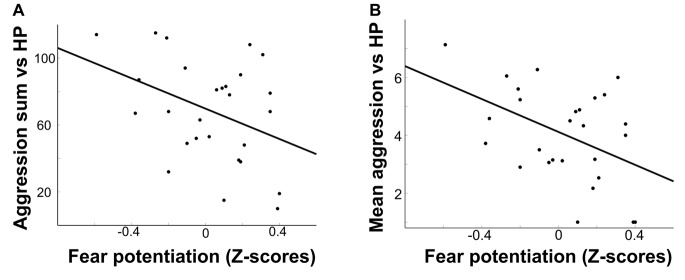
Scatterplots depicting the negative correlations between Fear Potentiation (FP) and aggression sum scores **(A)**, and between FP and mean aggression **(B)** against the highly provoking opponent (HP).

We found a negative correlation between FP and the summed punishment score for the provocative opponent (*r* = −0.38, *p* < 0.05; Figure 2A). This relationship was not significant for the non-provocative opponent (*r* = −0.28, *p* = 0.14). These correlations did not significantly differ from each other (*p* = 0.58). Concordantly, the difference in aggression sum scores between the provoking and non-provoking opponent was not correlated with FP (*p* = 0.44). We observed similar effects for mean aggression scores, with a negative correlation between FP and aggression against the provocative opponent (*r* = −0.44, *p* < 0.05; Figure 2B), but no effect for the non-provocative opponent (*r* = −0.17, *p* = 0.39). These two effects only differed marginally from each other, *t*_(26)_ = 1.8, *p* = 0.08. The difference in mean aggression between opponents was negatively correlated at trend level with FP (*r* = −0.35, *p* = 0.07). We found no relationship between FP and the number of times the avoidance option was chosen for either opponent or the absolute number of trials avoided (all *p* > 0.2). Mean aggression towards the provocative opponent was positively correlated with trait anger (*r* = 0.44, *p* < 0.05). There was a negative correlation between FP and trait anger as assessed with the AQ (*r* = −0.46, *p* < 0.05). Thus, FP was negatively related to trait anger, aggressive behavior towards the provocative opponent, as well as to a conglomerate measure of aggression and avoidance for this opponent. We observed no other relationships with self-report data (all *p* > 0.1).

### Discussion

In this experiment, we found a negative relationship between fear reactivity, as measured using a fear potentiated startle paradigm, and aggressive behavior towards a provocative opponent. In contrast to our previous study, where we found no such effect, participants here had the option of avoiding the aggressive interaction. Interestingly, we found no direct relationship between avoidance behavior and FP. The intrinsically low variability in avoidance (i.e., a maximum 10 times) might have curtailed the possibility to find direct relationships between avoidance and other parameters. Nonetheless, there was a negative relationship between FP and a conglomerate measure of aggression and avoidance against the provoking opponent. Thus, in situations where avoiding a confrontation is explicitly possible, participants high in fear reactivity behave less aggressively towards an aggressive opponent than participants low in fear reactivity. Note anyway that the relationship between aggression scores and FP did not significantly differ between opponents. Hence, FP might reflect general avoidant tendencies rather than reactivity to provocation specifically. Such tendencies seem to be slightly more pronounced when facing a provoking rival, but apply to interpersonal confrontation in general.

The negative correlation we found between FP and trait anger supports previous findings using startle paradigms which suggest that anger is an emotion related to behavioral approach tendencies (Amodio and Harmon-Jones, 2011). FP, on the other hand, is a defensive reflex associated with behavioral avoidance, like freezing behavior in animals (Davis et al., 1993). The positive correlation we found between trait anger and aggression towards the provocative opponent further underlines the role of emotional reactivity in aggressive retaliation. Thus, people high in fear reactivity are overall less prone to feelings of anger and are less likely to retaliate. People low in fear reactivity, on the other hand, report more angry impulses, which are related to more aggressive behavior. One must note, however, that we conducted correlations with personality questionnaires in an exploratory approach and the observed effects would not survive correction for multiple testing.

Our results might seem partly at odds with fMRI studies linking aggression with increased amygdala reactivity to threat (Coccaro et al., 2007; Carré et al., 2013; McCloskey et al., 2016). However, in a previous study from our group we implemented the FOE in the scanner and observed that the amygdala was recruited when avoiding a highly provoking opponent (Buades-Rotger et al., 2017). Thus, emotional reactivity to threat appears to favor either avoidance or aggression depending on threat escapability. Indeed, recent accounts of amygdala function posit that this structure generally codes for biologically relevant events, regardless of their aversive or appetitive nature (Pessoa and Adolphs, 2010; Weymar and Schwabe, 2016).

It is also intriguing that we did not find a clear FP effect, or a difference in avoidance between opponents. The lack of FP might be due to a failure in eliciting strong emotional reactions and/or to high interindividual variability in these affective responses (Bradley et al., 2001). We deem it unlikely that the white noise was itself too aversive, since we applied the same regime as in our previous study, where we did observe FP (Beyer et al., 2014). The absence of a provocation effect on avoidance may be attributable to the fact that the avoidance option only provided a momentary respite, as subjects could face the opponent they just avoided. Hence, in Experiment 2 we programed the task so that participants faced the opponents in alternating order, which should render the avoidance option more meaningful.

## Experiment 2

### Introduction

In our first experiment, we used FP as a measure of fear reactivity in order to compare our findings to our previous study using the TAP. This showed the proposed negative relationship between FP and aggression and supports our previous argument that the lack of an avoidance option in the TAP reduces interpersonal variability in aggression, as it imposes an unnatural limitation of behavioral options upon the participant. To further validate the paradigm and test the proposed negative relationship between avoidance and aggression, in a second experiment we combined the FOE-paradigm with two well-established tasks designed to measure implicit approach and avoidance tendencies towards social stimuli. Furthermore, we set task so subjects confronted each opponent alternatingly. We did so in order to potentiate the meaningfulness and salience of the avoidance option.

In the AAT (Roelofs et al., 2005), participants are asked to perform approach movements (i.e., pulling a joystick towards themselves) or avoidance movements (pushing a joystick away from themselves) in response to visual stimuli. Using stimuli that typically elicit approach or avoidance tendencies, congruency effects can be observed: participants are faster in pushing away stimuli that elicit avoidance tendencies than they are at pulling them close, whereas the opposite effect is observed for approach-related stimuli. This has been found for social stimuli as angry and happy faces (Roelofs et al., 2005), as well as other affective stimuli, as pictures of spiders in participants high in fear of spiders (Rinck and Becker, 2007). The AAT thus constitutes a measure of *behavioral* avoidance.

The DPT (MacLeod et al., 1986) assesses automatic orientation of attention towards one of two visual stimuli. In each trial, two stimuli are shortly presented at opposing sides of the screen center. After stimulus offset, a dot is presented on one side, and the participant is asked to press a corresponding response button (i.e., left or right). As individuals tend to initially allocate attention to threatening stimuli and then avoid them (Cooper and Langton, 2006; Rinck and Becker, 2006), they should be slower to respond to a dot presented on the side of the threatening stimulus at long exposition times (Mogg et al., 2010). The DPT can thus be used as a measure of *attentional* avoidance.

In this experiment, we used the AAT with pictures of angry and happy facial expressions and the DPT with pictures of angry and neutral facial expressions. We hypothesized that participants who showed high behavioral and attentional avoidance of angry faces would be less aggressive towards an aggressive opponent in the FOE-paradigm and would more frequently use the avoidance option for that opponent compared to participants showing less avoidance of angry faces.

### Materials and Methods

#### Participants and Procedure

Forty-two healthy volunteers (18 men; M_age_ = 22 ± 3 years) participated in this study. Participants were invited to the lab in same-sex triads. They were told that they would perform three computer tasks, the first of which would be interactive. The order of the two individual tasks was randomized across participants. After the computerized tasks, participants filled out the same questionnaires as in Experiment 1 (i.e., German versions of AQ, BIS/BAS and IRI, and the deception check). All participants were debriefed regarding the true study goals and reimbursed for their participation with 8 Euro per hour. This study was carried out in accordance with the recommendations of the University of Lübeck Ethics Commission (Ethikkommission der Universität zu Lübeck) with written informed consent from all subjects. All subjects gave written informed consent in accordance with the Declaration of Helsinki. The protocol was approved by the University of Lübeck Ethics Commission (Ethikkommission der Universität zu Lübeck).

#### FOE-Paradigm

The setup of the FOE-paradigm was identical to that used in Experiment 1, with one exception: instead of randomizing the trials against the two opponents, we now implemented an alternating order. Thus, participants knew that they would be playing against the two opponents alternatingly. We modified this in order to increase the salience of the avoidance option, as reasoned above. Whereas in Experiment 1, subjects could only minimize the absolute number of trials they played against the aggressive opponent, a trial where they chose to avoid this opponent could still be followed by a trial against the same opponent. As such, the avoidance was realized on a global level rather than immediately. To make the avoidance more prominent, the alternating schedule implemented here ensured that if a participant avoided the aggressive opponent, in the following trial they would always be playing against the non-aggressive one.

#### Approach-Avoidance-Task

For this task, we used photographs of angry and happy facial expressions of the Radboud Faces Database (Langner et al., 2010). We used pictures of 30 individuals (15 females), each showing an angry facial expression in one picture, and a happy expression in the other. Pictures of nine different individuals (four females) were used for practice blocks. The pictures were cropped into an oval shape, removing hair, ears and neck.

Participants were given a standard joystick (Speedlink^®^ Dark Tornado) as response device. At the beginning of each trial, a fixation cross was presented centrally on a white background. The participant started the trial by pressing the “shoot” button on the joystick. Following this, one picture was presented centrally on the screen. Participants could reduce picture size by pushing the joystick away from them. By pulling the joystick towards themselves, they could increase picture size. Picture size was varied gradually in seven steps.

In one block, participants were instructed to “push away” angry faces and “pull close” happy faces as quickly as possible; in the other block, the reverse instruction was given. Block order was randomized between subjects. Each block consisted of 30 happy and 30 angry trials, and the same pictures were used in both blocks. Each block was preceded by a practice run. During practice runs, each trial was followed by feedback (a green check-mark for correct reactions, a red cross for errors). The practice run for the first block consisted of 20 trials. The second practice run consisted of 28 trials, since participants had to reverse their response patterns from the first block.

For RT, we analyzed the interval between stimulus presentation and movement onset. Incorrect trials (including trials in which the initial movement was performed in the incorrect direction, followed by a correction), and trials with response latencies shorter than 150 ms or longer than 3 SD from the own mean were excluded from analysis. We calculated the pull minus push difference in RT (higher scores meaning higher avoidance) for angry and happy faces separately. We compared both biases with a paired *t*-test.

#### Dot-Probe-Task

For the DPT, we used 40 pictures from a set of previously validated videos (Kircher et al., 2013) showing angry and neutral facial expressions of 20 different professional actors (nine women). In each trial, the neutral and angry pictures of one person were presented together, to the left and right of the screen center.

Each trial began with a centrally located fixation cross being presented for a random interval between 500 ms and 1000 ms. Then, the neutral and angry pictures of one person were presented for 1000 ms. At picture offset, a dot was presented located to the left or right of the screen center, at the coordinate where the center of the respective picture had been. Participants were instructed to react as quickly as possible to the dot, by pressing a left button (A) on the computer keyboard if the dot was presented on the left, and a right button (L) if it was presented at the right side. We chose a presentation time of 1000 ms to allow sufficient time for eliciting avoidance, based on previous results showing that an avoidant bias can already be observed at 500 ms (Cooper and Langton, 2006; Pintzinger et al., 2017).

The task consisted of two blocks. In each block, 80 trials were presented, with each picture pair presented four times, once in each of the four conditions: angry picture on the left, dot probe on the left; angry picture on the left, dot probe on the right; angry picture on the right, dot probe on the right; and angry picture on the right, dot probe on the left.

We analyzed attentional approach vs. avoidance tendencies for angry pictures by subtracting mean RT for the neutral condition (dot probe in location of neutral picture) from angry conditions (dot probe in location of angry picture). The higher this score was for a given participant, the greater was this participant’s RT cost for reacting to a probe in the location of an angry picture. Thus, higher scores for this task represent greater attentional avoidance of angry facial expressions.

#### Statistical Analyses

We compared mean punishment selections and mean avoidance against the provoking vs. non-provoking opponent with paired *t*-tests, as in Experiment 1. We also compared approach vs. avoidance scores in the AAT, as well RTs for angry vs. neutral trials in the DPT. Additionally, we inspected whether men and women differed in their aggression and avoidance scores against each opponent with an analysis of variance (ANOVA) with provocation as within-subject factor and gender as between-subject factor.

To explore the relationship between avoidance of threatening social stimuli and behavior in the FOE-paradigm, we correlated behavioral scores of the aggression task (mean aggression scores for each opponent; summed avoidance scores for each opponent; conglomerate avoidance-aggression score for each opponent) with the RT scores of the AAT and the DPT. As in Experiment 1, significant correlations were compared between opponents with the *r.test()* function (Revelle, 2017). We also correlated self-report scores with behavioral measures from the task on an exploratory basis. Significance was again set at *p* < 0.05. Descriptive statistics for all measures in Experiment 2 are provided in Table 2.

**Table 2:** Descriptive statistics for questionnaires and computerized measures in Experiment 2 (*n* = 34).

	Mean ± SD
AQ total	56.94 ± 16.09
AQ physical aggression	18.11 ± 6.12
AQ physical aggression	13.44 ± 6.14
AQ verbal aggression	12.70 ± 2.68
AQ anger	12.67 ± 4.54
BIS	2.59 ± 0.51
BAS	3.19 ± 0.26
IRI fantasy scale	20.91 ± 3.44
IRI empathic concern	21.02 ± 2.00
IRI perspective taking	23.44 ± 2.75
IRI personal distress	16.44 ± 3.17
AAT angry pull—push (ms)	26.84 ± 44.09
AAT happy pull—push (ms)	−20.86 ± 67.01
DP congruent	364 ± 51
DP incongruent	365 ± 51
FOE avoidance HP	2.58 ± 2.20
FOE avoidance LP	1.73 ± 1.74
FOE aggression HP	4.14 ± 1.50
FOE aggression LP	3.82 ± 1.51

*AQ, Aggression Questionnaire; BIS, Behavioral Inhibition System; BAS, Behavioral Activation System; IRI, Interpersonal Reactivity Index; AAT, Approach-Avoidance Task; DPT, Dot-Probe Task; HP, High Provocation; LP, Low Provocation*.

### Results

Of the 42 participants, eight had to be excluded (five due to non-deception, one due to incomplete task data, two due to extreme bias scores of ±3 SD in AAT and DPT). Hence, analyses for this experiment were performed on 34 participants (13 men). Subjects reported the highest tone (*M* = 5.5, SE = 0.2) to be significantly more unpleasant than the lowest tone (*M* = 1.5, SE = 0.1), *t*_(33)_ = 13.9, *p* < 0.001.

**Figure 3:**
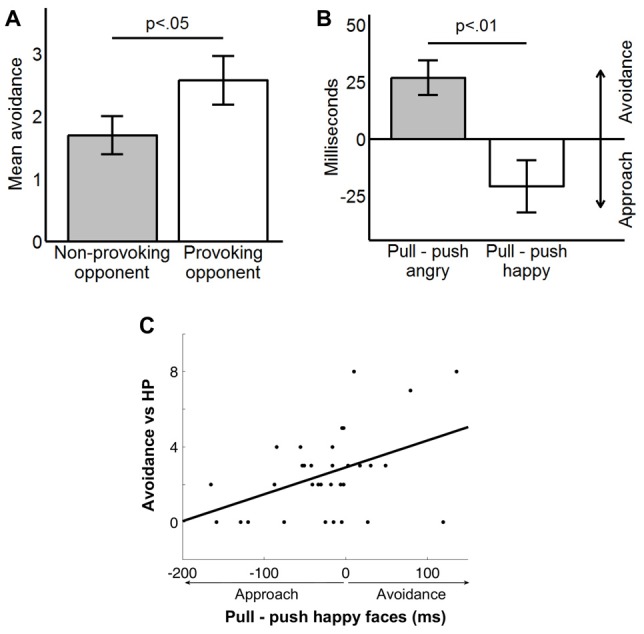
**(A)** Avoidant behavior against each opponent in the FOE. Values are mean ± standard error. **(B)** Results of the Approach-Avoidance Task (AAT). **(C)** Correlation between the approach bias in the AAT (pull minus push for happy faces) and avoidant behavior against the highly provoking (HP) opponent in the FOE.

Regarding behavior in the FOE paradigm, participants tended to select marginally higher punishments against the provoking opponent (*M* = 4.1, SE = 0.2) relative to the non-provoking one (*M* = 3.7, SE = 0.2), *t*_(33)_ = 1.9, *p* = 0.06. Crucially, they avoided the provoking opponent (*M* = 2.6, SE = 0.3) more often than the non-provoking one (*M* = 1.8, SE = 0.3), *t*_(33)_ = 2.1, *p* < 0.05 (Figure 3A). Participants used the avoidance option 4.3 times on average (SE = 0.5; range 0–10). The interaction between gender and provocation was non-significant for both aggression (*p* = 0.18) and avoidance (*p* = 0.55), indicating that behavior in the FOE did not differ between men and women.

We found the expected effect in the AAT, *t*_(33)_ = 2.8; *p* < 0.01 (Figure 3B), such that participants showed an avoidant bias for angry faces (*M* = 47 ms, SE = 14 ms) and an approach bias towards happy faces (*M* = −44 ms, SE = 20 ms). We did not observe the hypothesized avoidant bias in the DPT (*p* > 0.2), since RT were similar in neutral (*M* = 365 ms, SE = 8 ms) and angry trials (*M* = 364 ms, SE = 8 ms). DPT and AAT scores were uncorrelated (all *p* > 0.1).

The DPT or AAT biases for angry faces were not related to avoidance or aggression in the FOE (all *p* > 0.1). There was, however, a significant correlation between AAT scores for happy faces and avoidance against the provoking opponent (*r* = 0.43, *p* < 0.05). Namely, participants who were quicker to pull happy faces towards them, but were slower to push them away, avoided the provoking opponent less often (Figure 3C). The approach bias for happy faces was unrelated to avoidance of the non-provoking opponent (*r* = 0.01, *p* = 0.97). These two correlation coefficients differed significantly from each other, *t*_(33)_ = 2.23, *p* < 0.05, and, concordantly, the difference in avoidance between opponents was also associated with AAT approach scores (*r* = 0.39, *p* < 0.5). There were no correlations between self-report data and avoidance or aggression (all *p* > 0.1).

### Discussion

In this second experiment, we modified the task to make the avoidance option more meaningful, so that participants faced each opponent alternatingly, i.e., they could not face the same opponent in two consecutive trials. As intended, this caused participants to avoid the provoking opponent more than the non-provoking one. Participants selected slightly higher punishments against the provoking opponent than against the non-provoking one, but less so than in Experiment 1, indicating that subjects retaliated more evenly against both rivals. There were no gender differences in avoidance or aggression, suggesting that the provocation effect for avoidance observed in Experiment 2 is not due to the inclusion of male participants. Given that in Experiment 2 avoidance was a more attractive alternative strategy and the task was more predictable, participants might have experienced an increased sense of safety and confidence. This should favor the activation of appetitive, rather than defensive, motivational systems (Lang and Bradley, 2013). Hence, perhaps aggression in Experiment 2 reflected an appetitive drive, and not so much a defensive reflex. The results of correlational analyses, which are subsequently commented, support this account.

Instead of the expected relationship between an avoidant bias for angry faces and avoidance in the FOE, we found that participants with high approach scores towards happy faces engaged in more aggressive encounters in high provocation trials. Since happy faces constitute a social reward signal (Rademacher et al., 2010; Ruff and Fehr, 2014), our results indicate that individuals who are more strongly motivated by positive stimuli will tend to be less avoidant when provoked. This is consistent with the notion of aggression as an approach-related behavior (Carver and Harmon-Jones, 2009; Berkowitz, 2012). On the other hand, the fact that avoidance was related to AAT, but not to DPT scores, suggests that fight-vs.-flight decisions as implemented in the FOE are driven by general behavioral tendencies rather than implicit attentional biases.

The finding that AAT scores for happy rather than angry faces were related to avoidance deserves however further discussion. Some authors have argued that individuals showing an approach bias towards angry faces should be more aggressive, as they should be more prone to interpret anger expressions as a challenge rather than a threat, i.e., as an appetitive stimulus (van Honk et al., 2001; Beaver et al., 2008). However, happy faces are generally less ambiguous than angry faces (Coupland et al., 2004; Becker et al., 2011; Parmley and Zhang, 2015) and they more clearly convey reward and positive valence (Averbeck and Duchaine, 2009; Furl et al., 2012). Hence, happy facial expressions should more consistently elicit approach motivation than angry ones. In line with this formulation, and dovetailing our findings, a recent study using the AAT in veterans found that anxious symptomatology was related to avoidance of happy faces, but not to biases towards or away from angry expressions (Clausen et al., 2016). Similarly, another study with the AAT found that approach scores towards positive stimuli predicted reactive aggression, but no effect was found for angry faces (Lobbestael et al., 2016).

### General Discussion

Most established laboratory measures of aggression do not allow participants to avoid confrontation. We addressed this issue by developing and validating a version of the TAP that included an avoidance option: the FOE paradigm. In two separate experiments, we showed that reactivity to threat as measured by FP relates to reduced aggression and avoidance, and that participants with stronger approach tendencies towards positive stimuli more frequently chose to engage in an aggressive interaction than participants who tended to avoid positive stimuli.

In Experiment 1, participants with stronger FP responses were less aggressive on average in response to provocation. FP was also negatively related to aggression sum scores against the provoking opponent, which can be understood as a composite measure of avoidance and aggression. In our previous fMRI study, we found no relationship between threat reactivity and aggression in inescapable encounters (Beyer et al., 2014). Here, by giving participants the possibility to avoid confrontation, we observed the previously hypothesized negative correlation between threat reactivity and aggression. Nevertheless, we found no direct relationship between FP and avoidance in the FOE (i.e., number of avoidance options). This might be due to the fact that the avoidance option was not salient enough, as participants could face the highly provoking opponent in the trial after avoiding her.

In Experiment 2, we set the task so participants played against each opponent alternatingly instead of pseudo-randomly. In so doing, the avoidance option became more meaningful, and participants avoided the provoking opponent more often than the non-provoking opponent. Crucially, subjects showing a stronger avoidant bias for happy faces used the avoidance option against the provoking opponent more frequently. We found no relationship between the AAT avoidant bias for angry faces and avoidance in the FOE. DPT scores, which represent attentional avoidance of angry faces, were also uncorrelated with behavior in the task.

Taken together, our results suggest that aggressive behavior as implemented in the FOE is driven by approach motivation to engage in aggressive interactions. Participants who tended to react to threatening stimuli with strong defensive reflexes behaved overall less aggressively towards a provoking rival. This supports our initial theory that participants high in fear reactivity should behave less aggressively if given the opportunity to escape. On the other hand, participants with strong approach tendencies towards positive stimuli chose to confront the aggressive opponent more often. Likely, fight-vs.-flight choices in these participants were mainly driven by the prospect of potentially being able to retaliate against the aggressor, whereas participants low in behavioral approach preferred to withdraw from the aggressive interaction. These findings are in line with prominent theories of anger and aggression as driven by appetitive approach (Harmon-Jones, 2003; Carver and Harmon-Jones, 2009; Berkowitz, 2012).

### Limitations and Future Directions

A few limitations should be mentioned. First, the avoidance option is available only *during* the game and can be used several times. In real provocation situations one can only retreat once and usually before the proper physical confrontation ensues. Moreover, there are often alternative strategies to curb the provocateur. In future studies, one could implement friendly choices (e.g., praising the opponent or being punished in her stead), and/or an option to aggress proactively (e.g., preemptive punishments). This would allow for a more direct measurement of aggressive relative to prosocial tendencies than existing paradigms such the Help/Hurt Task (Saleem et al., 2015), or the Inequality Game (Klimecki et al., 2016), and would also better cover the range of possible responses to —or safeguards from— interpersonal provocation. Related to this point, our sample might have suffered from range restriction due to situation selection (Dijkman and Devries, 1987), as highly avoidant subjects are unlikely to volunteer for such a study in the first place, and our participants were all healthy young students. Our task should thus be further validated on samples preselected based on extreme approach and avoidance. Future work would also benefit from cross-validating the FOE with other tasks such as the PSAP. In that case, one could relate avoidance in the FOE with the number of times one uses the defensive strategy, and aggression with the number of times one uses the offensive strategy. Nevertheless, as stated in the “Introduction” section, both the defensive and aggressive options in the PSAP are costly, and therefore can be assumed to entail a conflict between approach and avoidance tendencies. An additional possibility would entail using more realistic measures of approach and avoidance, such as those based on interpersonal distance, which can be implemented physically (Perry et al., 2016) and/or in virtual reality environments (McCall and Singer, 2015; McCall et al., 2016).

## Conclusion

In the present article, we have provided validation of the FOE paradigm. The FOE is a competitive RT task in which participants can avoid the confrontation against either of two purported opponents: a provoking and a non-provoking one. We showed that subjects with higher reactivity to threat and lower approach motivation towards positive stimuli are less aggressive and more avoidant when taunted by a provocateur. Thus, the FOE can be readily implemented to measure not only retaliation intensity, but also subjects’ proneness to avoid or engage in aggressive encounters.

## Author Contributions

FB, MB-R, and UMK conceived the study and coordinated the experiments. FB, MB-R and MC performed the experiments. FB and MB-R analyzed the data and wrote the manuscript. MC and UMK revised the manuscript. All authors read and approved the manuscript.

## Conflict of Interest Statement

The authors declare that the research was conducted in the absence of any commercial or financial relationships that could be construed as a potential conflict of interest.
